# Health effects after inhalation of micro- and nano-sized zinc oxide particles in human volunteers

**DOI:** 10.1007/s00204-020-02923-y

**Published:** 2020-10-01

**Authors:** Christian Monsé, Monika Raulf, Birger Jettkant, Vera van Kampen, Benjamin Kendzia, Leonie Schürmeyer, Christoph Edzard Seifert, Eike-Maximilian Marek, Götz Westphal, Nina Rosenkranz, Rolf Merget, Thomas Brüning, Jürgen Bünger

**Affiliations:** Institute for Prevention and Occupational Medicine of the German Social Accident Insurance, Institute of the Ruhr-Universität Bochum (IPA), Bürkle-de-la-Camp-Platz 1, 44789 Bochum, Germany

**Keywords:** Deposition efficiency, Human inhalation study, Micro-sized particles, Nanoparticles, Zinc oxide

## Abstract

Inhalation of ZnO particles can cause inflammation of the airways and metal fume fever. It is unclear if different sizes of the particles alter these effects. However, various studies report higher biological activity of other nano-sized particles compared to microparticles. No effects at all were observed after inhalation of micro- and nano-sized zinc oxide (ZnO) particle concentrations of 0.5 mg/m^3^. Studies with different particle sizes of ZnO at higher exposures are not available. Accordingly, we hypothesized that inhalation of nano-sized ZnO particles induces stronger health effects than the inhalation of the same airborne mass concentration of micro-sized ZnO particles. 16 healthy volunteers (eight men, eight women) were exposed to filtered air and ZnO particles (2.0 mg/m^3^) for 2 h (one session with nano- and one with micro-sized ZnO) including 1 h of cycling at moderate workload. Effect parameters were symptoms, body temperature, inflammatory markers in blood and in induced sputum. Induced sputum was obtained at baseline examination, 22 h after exposure and at the end of the final test. The effects were assessed before, immediately after, about 22 h after, as well as two and three days after each exposure. Neutrophils, monocytes and acute-phase proteins in blood increased 22 h after micro- and nano-sized ZnO exposure. Effects were generally stronger with micro-sized ZnO particles. Parameters in induced sputum showed partial increases on the next day, but the effect strengths were not clearly attributable to particle sizes. The hypothesis that nano-sized ZnO particles induce stronger health effects than micro-sized ZnO particles was not supported by our data. The stronger systemic inflammatory responses after inhalation of micro-sized ZnO particles can be explained by the higher deposition efficiency of micro-sized ZnO particles in the respiratory tract and a substance-specific mode of action, most likely caused by the formation of zinc ions.

**Electronic supplementary material:**

The online version of this article (10.1007/s00204-020-02923-y) contains supplementary material, which is available to authorized users.

## Introduction

Employees exposed to zinc-containing fumes may suffer from metal fume fever after inhalation, especially during handling of hot-dip galvanized sheet metal or when welding galvanized steel. Typical symptoms are dyspnea, fever, and flu-like symptoms occurring with a latency period of about 4–12 h and resolving spontaneously within 48 h (Nemery 1990).

Human experimental inhalation studies using ZnO particles are sparse—particularly with different particle sizes. While a number of older experimental inhalation studies reported adverse health effects after high exposures to zinc oxide (ZnO) (Gordon et al. 1992; Fine et al. 1997), no effects were detected in 12 subjects after inhalation of 0.5 mg/m^3^ ZnO for 2 h at rest (Beckett et al. 2005). In the latter study, participiants were exposed to different ZnO particle sizes (micro- vs. nano-sized). However, due to the general absence of effects after inhalation, it was also not possible to demonstrate different effects between the particles sizes.

Several recent studies focused on exposures of zinc-containing welding fumes in various settings (different concentrations and inhalation times, repetitive inhalations). The key aspect of these studies was a simulation of exposure conditions close to the workplace, but the particle size was not experimentally modified (Brand et al. 2014, 2019; Krabbe et al. 2019).

In a previous study, we were able to demonstrate a concentration–response relationship between ZnO nano-sized particles and systemic effects (Monsé et al. 2018). Whereas no relevant effects were detectable after sham exposures and 0.5 mg/m^3^ ZnO, reversible systemic effects on acute-phase proteins (C-reactive protein (CRP), serum amyloid A (SAA)) and neutrophils in blood occurred after ZnO exposure with 1.0 and 2.0 mg/m^3^ for 4 h. Clinical effects were strongest after 2.0 mg/m^3^ ZnO, with flu-like symptoms and elevated body temperature in several subjects. The assessment of local effects in the same subjects (Monsé et al. 2019) yielded reversible concentration-independent changes of inflammatory parameters in the airways after inhalation of ZnO in the range from 0.5 to 2.0 mg/m^3^. The lowest concentration did not induce metal fume fever or significant inflammatory systemic effects (Monsé et al. 2018).

Until now, it is unclear whether different particle sizes lead to different effect strengths in humans at the same airborne ZnO mass concentration. We hypothesized that inhalation of nano-sized ZnO particles induces stronger inflammatory responses than micro-sized particles according to animal experiments conducted with respirable granular biodurable particles (GBP) (Gebel 2012; Oberdörster et al. 1994). The same effect could also be expected from nano-sized ZnO due to its faster dissolution behaviour and/or larger surface area.

## Materials and methods

### Micro-sized ZnO particles

A self-constructed nebulizer was installed in the air conditioning duct of the exposure unit at our institute (Monsé et al. 2012), equipped with a 7 L stirred tank and a self-priming two-substance nozzle (model 970, Düsen-Schlick GmbH, Untersiemau, Germany). A suspension of 12.0 g of purchased ZnO (Zinkweiss Harzsiegel CF, CAS No. 7440-66-6, Norzinco GmbH, Goslar, Germany) in 5.0 L of water (water purification system, model Milli-Q Advantage A 10, Merck KGaA, Darmstadt, Germany) was nebulized with pressurized air at 3.0 bar. The ZnO was sieved for 5 min before use, using the < 100 μm fraction (Vibratory Sieve Shaker, model AS 200 control, Retsch GmbH, Haan, Germany) to exclude larger clumps in the stirred tank. The suspension was stirred during dosing (260 rounds per minute). Two flow baffles were installed in the tank to ensure a turbulent flow. The metering of the ZnO was controlled via a pulse width modulation by means of a compressed air shutdown of the two-substance nozzle. The aerosol droplets of the sprayed suspension completely dried out during the flight into the exposure unit and released the desired ZnO particles.

### Nano-sized ZnO particles

The principle of the particle synthesis was based on the pyrolysis of atomized aqueous zinc formate solutions with a flame generator (Flammengenerator, model FG 3, MoTec Konzepte, Bochum, Germany) which was installed in the air conditioning duct. The diameter of the generated primary particles was about 10 nm. These particles formed aggregates and agglomerates with a mean of nearly 80 nm at a ZnO concentration of 2.0 mg/m^3^. A ceiling fan was used to homogenize the freshly generated ZnO nanoparticle atmospheres in the exposure unit (Pillar et al. 2016). Further details were published earlier (Monsé et al. 2018).

Briefly, constant target concentrations of 2.0 mg/m^3^ micro- and nano-sized ZnO were planned. Sham exposures (0 mg/m^3^ ZnO) were performed with filtered and conditioned air. All exposure scenarios were carried out with an air exchange rate at 12 per h (360 m^3^/h) with a room temperature of 24.3 °C (± 0.7 °C) and a relative humidity of 48.1% (± 4.3%).

### Characterization of ZnO particles

**Fig. 1:**
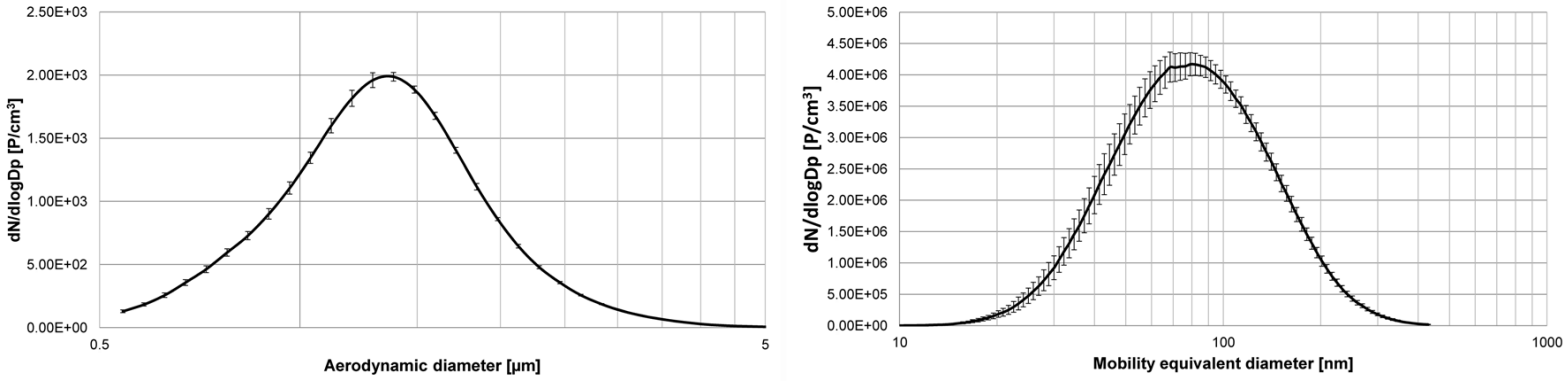
Averaged particle number and size distributions of airborne ZnO particles at 2.0 mg/m^3^. Left: micro-, right: nano-sized ZnO particles. In addition, the error bars of each individual size channel are shown

An Aerosol Particle Sizer (APS, model 3321, TSI Inc., Shoreview MN, USA, equipped with a 1:20 aerosol diluter, model 3302 A, TSI Inc.) measured the micro-sized particle size distributions. A Scanning Mobility Particle Sizer (SMPS, model 3080, TSI Inc., Shoreview MN, USA, equipped with a long differential mobility analyzer and a butanol condensation particle counter, model 3776, TSI Inc.) measured the nano-sized particle size distributions in the exposure unit. Both analyzers determined the number concentration and size distributions every 5 min. Figure 1 shows the particle size distributions of both ZnO particle fractions.

The curves represent an average of all days of the 2 h exposures at 2.0 mg/m^3^. There were negligible differences between targeted and measured concentrations (Table 1). The particle size distributions were both monomodal with relatively small geometric standard deviations of 1.71 (nano-sized) and 1.43 (micro-sized).

**Table 1:** Measured parameters of ZnO particles

Target concentration	Measured concentration	Mean particle diameter	Geometric standard deviation	Particle number concentration	Surface area (BET)
[mg/m^3^]	[mg/m^3^]	[nm]	[GSD]	[#/cm^3^]	[m^2^/g]
0.0	0.037 (± 38.1%)	–	–	< 500	–
2.0 nano-sized	2.017 (± 0.2%)	SMPS: 78.8 (± 4.1%)	1.71	2.58E + 06 (± 4.2%)	20.2
2.0 micro-sized	2.035 (± 0.4%)	APS: 1330 (± 1.0%)	1.43	1450 (± 2.5%)	4.8

Mass concentration measurements of airborne ZnO were recorded at 1-min intervals using a tapered elemental oscillating microbalance (TEOM, model 1400a, Rupprecht and Patashnik, Albany NY, USA). Both the airborne mass of ZnO particles and the drying process of the ZnO suspension were confirmed by gravimetric measurement using total dust sampling systems (model GSP, GSA Messgerätebau GmbH, Ratingen, Germany) with cellulose nitrate filters (Sartorius Stedim Biotech GmbH, 8 µm pore size, 37 mm diameter). The filters were exposed with a volume flow of 10.0 L/min. for 2.5 h (air sampler, model SG10-2, GSA Messgerätebau GmbH, Ratingen, Germany). The deviation between the TEOM and gravimetric measurements was smaller than 2.5% each.

Trace gas analyses of nitric oxides (NO, NO_2_) were performed via online chemical ionization mass spectroscopy at 1-s intervals (CIMS, model Airsense.net, MS4 GmbH, Rockenberg, Germany) to control the pyrolysis process. Further details are given by Monsé et al. (2014).

The specific surface area was determined using a BET device (BET, model Gemini VII 2390a, Micromeritics GmbH, Aachen, Germany). Nano-sized ZnO particles were deposited by thermophoresis on a quartz glass tube placed over the flame cone of the flame generator.

All measurement results of airborne ZnO concentrations, mobility particle diameter, geometric standard deviations, particle number concentration (averaged over all exposure days for each exposure condition) as well as specific surface area are listed in Table 1.

The morphology of the particles on a SEM (scanning electron microscopy) pin stub was characterized by scanning electron microscopy (SEM, model JSM-7500F, JEOL Ltd., Tokyo, Japan) with a nominal resolution of 2 nm.

**Fig. 2:**
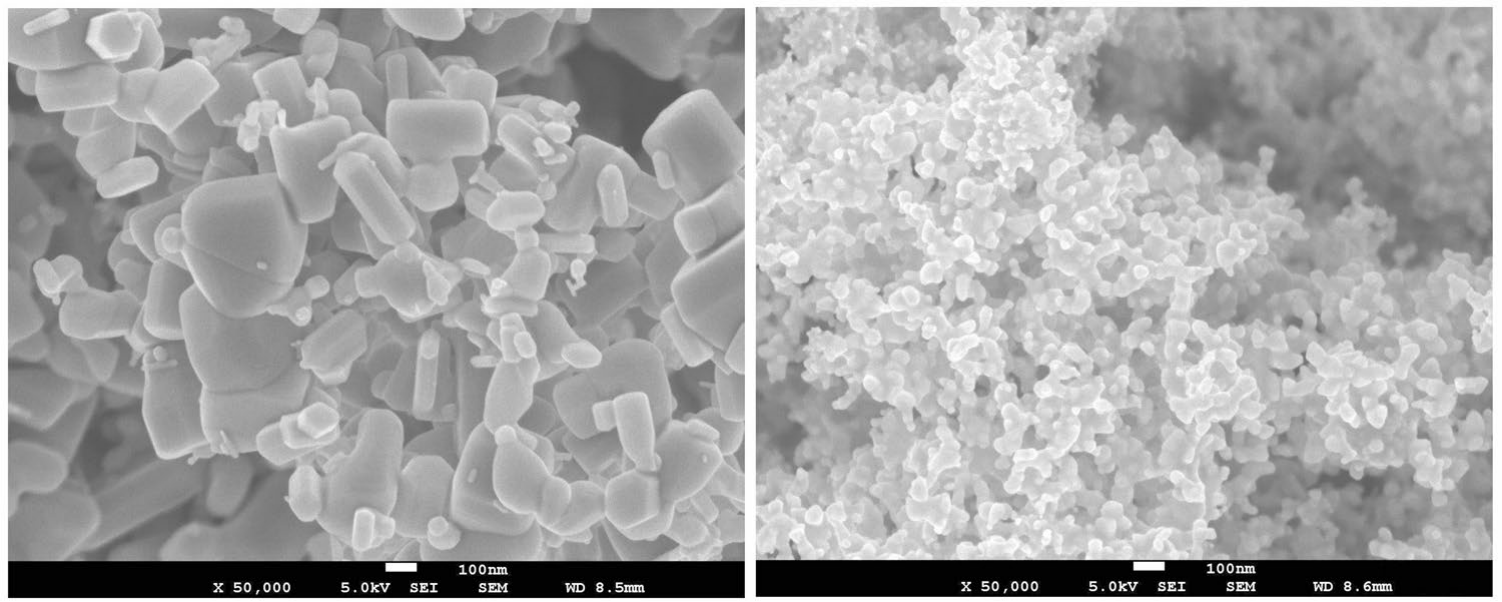
SEM (scanning electron microscopy) pictures of ZnO particles. Left: micro-, right: nano-sized ZnO particles at a magnification of 50,000 each

The left image in Fig. 2 shows the micro-sized ZnO particles which consisted of individual crystals. These particles were taken directly from the chemical packaging. The right image shows the ZnO nano-sized particles deposited by thermophoresis on a quartz glass tube placed over the flame cone of the flame generator. The primary ZnO particles with an approx. diameter of 10 nm formed larger aggregates and agglomerates.

X-ray powder diffraction of both ZnO particle fractions was determined using a diffractometer from Stoe with a Bragg–Brentano geometry (XRD, model Stadi P with Co Anode and scintillation counter, Stoe & Cie GmbH, Darmstadt, Germany). Comparing this with standard data, it was observed that all peaks matched the standard data of hexagonal phase of ZnO (JCPDS card no. 36-1451).

An elementary analysis of the nano-sized ZnO particles (Mikroanalytisches Labor Pascher, Remagen, Germany) yielded a purity of 99.7%. According to the manufacturer of the micro-sized ZnO, the purity was between 99.7 and 99.9%.

### Participants

Sixteen healthy non-smoking volunteers (eight women, eight men) with a median age of 26 (range 20–36) years participated in the study (Table 2). The subjects had no previous exposure to airborne zinc compounds and did not show bronchial hyperresponsiveness to methacholine as assessed with a reservoir method (Merget et al. 2005). The study participants had to be able to produce sputum according to our criteria (eosinophils < 1%, epithelial cells < 95% and neutrophils not dominant) to exclude subjects with airway inflammation for other reasons and to make sure that the material originated from the lower airways. Standard baseline laboratory parameters were within normal ranges. Four subjects (two women, two men) with specific IgE antibodies (sIgE) to ubiquitous aeroallergens (atopy screen sx1, Phadiatop, ImmunoCAP system, ThermoFisher Scientific, Phadia AB, Uppsala, Sweden) without any clinical manifestation were included, but their ZnO exposures were performed outside of the pollen season.

**Table 2:** Characteristics of the study subjects

Subjects	Total, *n* = 16	Male, *n* = 8	Female, *n* = 8
Age [years]	26 (20–36)	26 (21–36)	26 (20–29)
Height [cm]	176 (162–189)	182 (175–189)	168 (162–177)
Weight [kg]	73 (53–92)	81 (74–92)	62 (53–71)
Body mass index [kg/m^2^]	24 (19–28)	25 (23–28)	23 (19–24)
Total IgE [kU/L]	23 (9–287)	47 (10–287)	21 (9–83)
sIgE to sx1 [kU_A_/L]	0.11 (0.05–11.4)	0.10 (0.05–11.4)	0.12 (0.06–6.56)
sIgE to sx1 [n ≥ 0.35 kU_A_/L]	4	2	2

Medians and ranges are listed. Specific IgE (sIgE) to sx1 ≥ 0.35 kU_A_/L is an indicator of sensitization to environmental allergens

*BMI* body mass index

### Study design

**Fig. 3:**
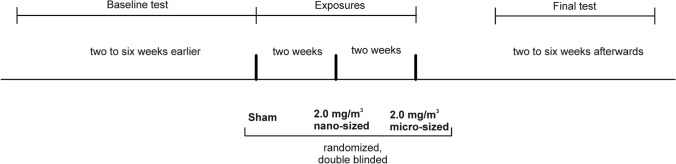
Time line of this study

The administered dose of ZnO was derived from the study of Monsé and co-workers (Monsé et al. 2018) to yield mild systemic inflammatory responses as assessed by symptoms, body temperature and inflammatory markers in blood and induced sputum. Subjects were exposed according to the scheme for 2 h (Fig. 3), with 2-week intervals between each exposure. The subjects were at rest except for two periods of 30 min with moderate physical activity individually set to 15 L/min/m^2^ (corresponding to a work load of about 60 watts (range 30–96 watts)) on a cycle ergometer. Exposures were randomized and double-blinded. Medical examinations were performed before, directly after, approximately 22 h after the start of exposure, as well as two and three days after the exposures.

Baseline testing was performed before the first exposure. The following examinations were done: detailed questionnaire-based medical history, physical examination, blood sampling, sputum induction and analysis, lung function testing, cotinine measurement in urine, measurements of fractional exhaled nitric oxide (FeNO), electrocardiogram, blood pressure, spiroergometry to assess the work load, methacholine testing and body temperature. After the last exposure, a final testing was performed including physical examination, blood and induced sputum sampling and analysis, electrocardiogram, blood pressure, lung function testing and body temperature. In addition, vital functions (electrocardiogram, blood pressure) were monitored during the exposures, which were always carried out from 10 to 12 am. Time of sample collection was recorded to adjust for possible diurnal varations in the biomarker levels.

**Fig. 4:**
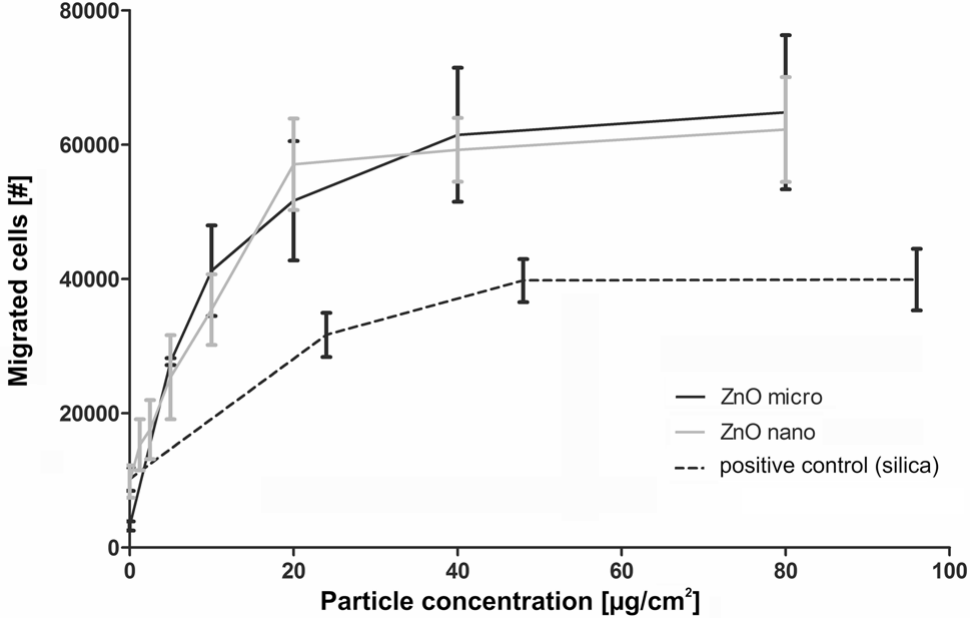
Chemotaxis (migrated cells) of the dHL-60 cells in response to supernatants of NR8383 cells challenged with micro- and nano-sized ZnO particles. Results represent arithmetic means and standard deviations of three independent experiments. Commercially available silica nanoparticles served as positive control

### Particle-induced cell migration assay (PICMA)

To assess whether the two different ZnO species differ in their acute biological effects, we analyzed the chemotactic attraction of differentiated human leukemia cells (dHL-60 cells) in response cell supernatants of particle challenged NR8383 rat alveolar macrophages, as a model for the particle-induced accumulation of neutrophils in the inflamed lung. The dHL-60 cells have properties similar to that of physiological neutrophil granulocytes. A detailed description of this functional assey was published before (Westphal et al. 2015). Briefly, we challenged the macrophages with both ZnO species separately for a period of 16 h. The cell supernatants were used to investigate migration of dHL-60 cells. A silica (SiO_2_) reference sample (CAS No. 7631-86-9, Lot MKBF2889V, 99.5%, 10–20 nm, Sigma-Aldrich, Steinheim, Germany) was used as positive control (Fig. 4). Further information is given in the supplementary material (PICMA).

### Questionnaire

All subjects answered a questionnaire addressing flu-like symptoms (at least one of three symptoms: feeling of fever, feeling sick and muscle pain) and airway irritation (throat irritation and/or cough) at different time points (before exposure, directly after exposure, 22 h after exposure as well as two and three days after exposure). To avoid any information bias, we added questions about clearing throat, shortness of breath, fatigue, headache, feeling warm, discomfort, chills, and feeling unwell. All symptoms were graded according to severity [not at all (0 score point), barely (1 points), little (2 points), moderate (3 points), strong (4 points), very strong (5 points)]. Sum scores and percentage of sum scores were generated for each study participant, previously described in detail (Monsé et al. 2019).

### Body temperature

Subjects measured their own body temperatures using a digital thermometer (model MT3001, Microlife AG, Widnau, Switzerland) before, during and after ZnO exposure and additionally every 2 h until the next day, but not during sleep. All participants were instructed to put the thermometer at minimum for 1 min under the tongue (sublingual) with the mouth closed and not to drink or eat 5 min prior to measurements. Nearly all circadian temperature fluctuations were inside 0.8 °C and lower than 37.5 °C, the limit considered as fever with an 0.1 °C error of measurement.

### Blood parameters

Inflammatory markers (differential blood cell count, CRP and SAA) were analyzed using standard methods. The total and differential blood cell counts were determined using the Coulter counter method with UniCell DxH800 (Beckman Coulter Inc., Brea CA, USA). ELISA techniques were used to quantify the following serum biomarker: club cell protein (CC16) (BioVendor Instruments, Brno, Czech Republic, range 1.57–50 ng/mL), SAA (Invitrogen™ Carlsbad, CA, USA; detection of human serum amyloid A1 cluster (Hu SAA) in the range of 9.4–600 ng/mL), and CRP (high-sensitive ELISA from IBL International, Hamburg, Germany; range 0.4–19 µg/mL).

Further standard clinical parameters of renal and liver function were determined during the recruiting process of the subjects.

### Induced sputum

Sputum samples were obtained at the baseline examination, 22 h post exposure and at the final examination, but not directly before exposures. This procedure eliminates the possibility that repeated sputum recovery within a short time period may induce inflammatory effects triggered by sputum induction itself. According to the procedure used in several studies (Raulf et al. 2015, 2017; Monsé et al. 2019), sputum induction was carried out by inhalation of nebulized isotonic saline solution (0.9% sodium chloride (NaCl); Pariboy, Pari GmbH, Weilheim, Germany) for 15 min. Concentrations of interleukin-8 (IL-8), matrix metalloproteinase-9 (MMP-9) and tissue inhibitors of metalloproteinases-1 (TIMP-1) were determined in the appropriate immunoassays based on monoclonal or polyclonal antibodies (Pharmingen, Heidelberg, Germany, Assay Design and/or Bio Vendor, all: Heidelberg, Germany) according to the recommendations of the manufacturers. The total protein determination was carried out with bovine serum albumin as a standard with a measuring range of 10 to 100 mg/L (Bradford 1976). The respective lower quantification limit was 3 pg/mL for IL-8, 31.2 pg/mL for MMP-9 and 9.76 pg/mL for TIMP-1.

### FeNO

Fractional exhaled nitric oxide (FeNO) was measured using a portable electrochemical analyzer (NIOX Mino, Aerocrine, Solna, Sweden) taking into account the guidelines of the American Thoracic Society (ATS 1995) and European Respiratory Society (ATS/ERS 2005).

### Lung function testing

Lung function was recorded using both body plethysmography (Crieé et al. 2011) and spirometry (ATS 1994) in a linked maneuver with a MasterLab (Vyaire Medical GmbH, Höchberg, Germany) according to ATS and ERS guidelines.

### Data analysis of effect parameters

Characteristics of subjects were expressed as medians as well as minimum and maximum (see Table 2). Descriptive analysis was performed for each variable stratified by exposure (sham, micro- and nano-sized 2.0 mg/m^3^ ZnO) and time of measurement (before, after 22 h as well as two and three days after exposure). Graphical representations were illustrated with boxplots. Effects were compared between before and 22 h after exposure. In addition, the effects after 22 h post exposure were compared between micro- and nano-sized ZnO particles. Exposure groups were compared using paired Student’s *t* test for normal or log-normal distributed variables. The problem of multiple comparisons was counteracted using the Bonferroni correction (Bonferroni 1936). Individual descriptive analyses were performed for body temperature with a cut-off of ≥ 37.5 °C. Differences in the blood and sputum parameters between different ZnO exposures and time of measurements were examined using multivariate generalized estimating equations (GEE) logistic regression (Liang and Zeger 1986). Here, we compared the differences for each parameter separately for the time of measurement and ZnO exposure using odds ratios (OR) and 95% confidence intervals (CI). These analyses were adjusted for BMI and total IgE.

### Estimation of lung deposition efficiency

To estimate the ZnO particle lung deposition efficiency, we modified the open-source code (Guha et al. 2014) based on the International Committee for Radiological Protection (ICRP) Publication 66 (ICRP 1994). In the first step, we measured the activity in dependence of breathing parameters of each subject. During the exposure with ZnO particles, we set the individual breathing flow rate per body surface area (DuBois and DuBois 1916) to 15 L/(min × m^2^) under cycling conditions and about 6 L/(min × m^2^) at rest. The cycling parameters and the respiratory rate together with the heart rate were continuously recorded during all 30 min rest and cycling sections (model Somno Screen Plus, SOMNOmedics, Randersacker, Germany). The flow rate, the tidal volume, the respiratory rate and heart rate were previously measured with a mobile spiroergometry system (model OxMobile with ECG, vyaire, Höchberg, Germany) at rest (sitting) and during a time period of 15 min cycling at constant load. Additionally, the increase or decrease of these parameters before and after cycling was taken into account. These time-dependent individual flow rates and the tidal volumes were taken as input to the lung deposition model. In the second step, we used the SMPS and APS particle size distribution measurements providing the quantity of particles between 10 nm and 10 µm diameter, assuming a spherical shape and constant material density of 5.61 g/cm^3^. Measurements with a TEOM device yielded the airborne particle mass in mg/m^3^. The absolute particle concentration distributions were calculated based on these information. We fitted normalized log-normal probability density functions of aerodynamic particle diameters replacing the proved normal distribution of monodisperse aerosols in the original source code. In the last step, we run the model assuming nose breathing with 30% proportion breathing through mouth. This resulted in particle depositions in the respiratory tracts of each individual in our study, subdivided in the alveolar, tracheobronchial and extrathoracic region. The mean and standard deviation of the incorporated masses were calculated for these three regions at different generated particle sizes (Statistica 13, Tulsa, US). Summing the time-dependent product of the recorded airborne particle mass and the individual minute volume of ventilation of each subject yielded the total ZnO mass intake.

### Ethics

The study was performed in accordance with the Declaration of Helsinki for Human Research. It was approved by the local Ethics Committee of the Ruhr University Bochum, Germany (No. 16-5910). All study participants gave written informed consent and received a financial compensation for participation.

## Results

### Particle-induced cell migration assay

Challenge of NR8383 cells with the nano- and micro-sized ZnO particles yielded cell supernatants that acted chemotactically towards dHL-60 cells in an almost identical way (Fig. 4). The comparison with the silica-positive control allows the conclusion that ZnO showed significantly stronger chemotactic effects than silica.

### Questionnaire

The evaluation of parts of the questionnaires relevant for this study (feeling of fever, feeling sick, muscle pain, throat irritation and/or cough) did not demonstrate an increase of the effect ratings after micro- and nano-sized ZnO exposure compared to sham. On average, the relative symptom sum score for all questions at each time point was 4.4%. The highest relative sum score of 12.5% was found for “throat irritation and/or cough” 22 h after nano-sized ZnO exposure. No significant differences were observed between micro- and nano-sized ZnO exposures.

### Body temperature

Except for one female subject who measured fever (38.1 °C) and reported general discomfort and scratching in the throat in the evening after her sham exposure, no increase of body temperature (≥ 37.5 °C) was observed after sham. One male subject exposed to micro-sized ZnO particles measured an increased temperature of 38.9 °C and one female participant had an increased temperature (37.9 °C) after exposure to ZnO nanoparticles. Both temperature increases occurred 18 to 20 h after the ZnO exposure.

### Blood parameters

**Fig. 5:**
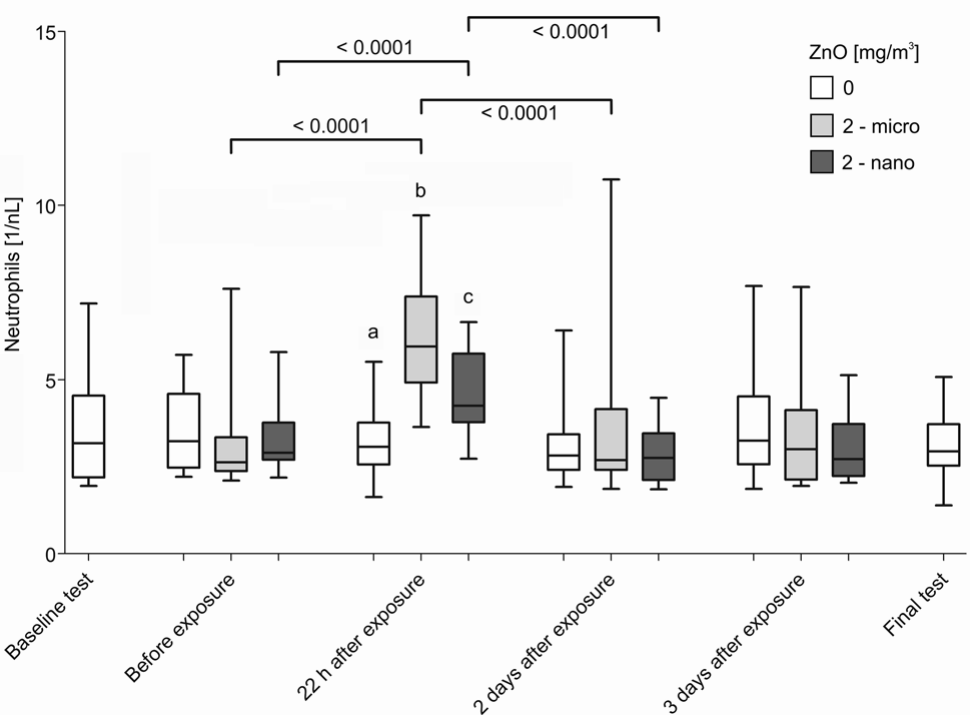
Neutrophil numbers in blood after sham (0 mg/m^3^ ZnO) or inhalation of micro- and nano-sized ZnO particles at the different time points. Differences between ab, ac and bc were all *p* < 0.0001. All differences *p* < 0.05 are shown. Significance levels *α* = 0.0055 (for brackets) and 0.0166 (for *a*, *b*, *c*) after Bonferroni correction

Figure 5 shows the univariate evaluation of the time course of neutrophil numbers. Further selected parameters in blood are described in the text (monocytes, CRP, SAA and CC16). All other blood cell counts showed no significant changes at any time (lymphocytes, thrombocytes, erythrocytes, eosinophils and basophils). The detailed results of all blood cell counts are presented in the supplementary material (Table S1).

The neutrophil numbers obtained from six examinations without ZnO exposure (baseline tests, examinations done before each exposure including sham, 22 h as well as two and three days after exposure and from final tests) were not significantly different from one another. This was also true for all other effect parameters in blood. Neutrophil levels increased 22 h after exposure to nano- and micro-sized ZnO compared to levels before the exposure (Fig. 5). 22 h after exposure, the increase of neutrophils was more pronounced after the micro-sized ZnO particle exposure. Two days after exposure, neutrophils had returned to levels within the range of the initial values.

Monocyte numbers increased significantly 22 h after the micro-sized ZnO exposure (*p* = 0.0004) compared to pre-exposure levels. Micro-sized ZnO showed a tendency to a higher increase compared to nano-sized ZnO (*p* = 0.0562). For all other time points, no differences were seen compared to levels before exposures.

Nano- and micro-sized ZnO exposures induced increases of CRP and SAA 22 h after exposure compared to pre-exposure levels. However, this effect was not significant for SAA after micro-sized ZnO exposure. Significant differences between micro- and nano-sized ZnO exposures were not observed for these acute-phase proteins. Two days after ZnO exposure, CRP and SAA were still elevated compared to pre-exposure levels. Three days after both ZnO exposures, CRP and SAA values decreased, but did not return to the level of the initial values until the final examination.

CC16 levels increased significantly 22 h after both ZnO exposures compared to pre-exposure levels. The difference in the increase of CC16 levels between nano- and micro-sized ZnO particles after 22 h was also statistically significant. The increase was more pronounced after the micro-sized ZnO particle exposure. Two days after both ZnO exposures, the CC16 levels decreased below the pre-exposure values and remained decreased also three days after the exposures. At the time of the final examination, the CC16 levels returned to the level of the baseline examination.

Table 3 shows the results of the GEE analyses for all blood parameters that showed an effect after ZnO exposures. A GEE analysis with the acute-phase protein SAA did not converge due to the large variance (< 1880–253,235) and was, therefore, not included in the table. The detailed results of all blood cells are presented in the supplementary material (Table S2).

**Table 3:** GEE analyses of selected blood parameters considering ZnO particle size and time of sampling

Parameter	Time of sampling	ZnO [mg/m^3^]	OR^a^	OR 95% CI	*P* value
Neutrophils [1/nL]	Before exposure	0	1.00		
	22 after exposure	2–micro	2.11^#^	1.71–2.60	** < 0.0001**
	22 after exposure	2–nano	1.57^#^	1.37–1.80	** < 0.0001**
	2 days after exposure	2–micro	1.17	0.92–1.49	0.2070
	2 days after exposure	2–nano	0.99	0.82–1.19	0.8826
	3 days after exposure	2–micro	1.05	0.85–1.28	0.6552
	3 days after exposure	2–nano	0.92	0.76–1.13	0.4388
	Final examination		1.02	0.82–1.24	0.8376
Monocytes [1/nL]	Before exposure	0	1.00		
	22 after exposure	2–micro	1.32	1.15–1.52	** < 0.0001**
	22 after exposure	2–nano	1.16	1.01–1.34	**0.0337**
	2 days after exposure	2–micro	1.04	0.85–1.27	0.7105
	2 days after exposure	2–nano	1.02	0.88–1.18	0.7842
	3 days after exposure	2–micro	0.97	0.81–1.17	0.7603
	3 days after exposure	2–nano	0.94	0.80–1.12	0.5013
	Final examination		1.13	0.99–1.28	0.0601
CRP [µg/mL]	Before exposure	0	1.00		
	22 after exposure	2–micro	1.95	1.04–2.88	**0.0348**
	22 after exposure	2–nano	1.65	1.01–2.29	**0.0479**
	2 days after exposure	2–micro	2.45	1.07–3.81	**0.0023**
	2 days after exposure	2–nano	1.47	0.88–2.35	0.4111
	3 days after exposure	2–micro	1.52	0.89–2.41	0.3857
	3 days after exposure	2–nano	0.90	0.77–1.06	0.2532
	Final examination		1.23	0.64–2.17	0.5247
CC16 [ng/mL]	Before exposure	0	1.00		
	22 after exposure	2–micro	1.12	1.00–1.25	**0.0455**
	22 after exposure	2–nano	1.07	0.97–1.19	0.1828
	2 days after exposure	2–micro	0.79	0.73–0.85	** < 0.0001**
	2 days after exposure	2–nano	0.82	0.74–0.90	** < 0.0001**
	3 days after exposure	2–micro	0.77	0.67–0.89	**0.0003**
	3 days after exposure	2–nano	0.82	0.72–0.93	**0.0021**
	Final examination		0.88	0.78–1.00	0.0524

^a^OR adjusted for ZnO exposure, time of sampling, BMI, and total IgE. *P* values in bold are statistically significant

^#^Statistically significant difference in effect size between micro- and nano-sized ZnO particles

All adjusted OR were higher for the time point 22 h after both ZnO exposures compared to pre-exposure time points. OR of pre-exposure levels were restored at the final examination of all study participants. For all parameters, the increases of OR were more pronounced 22 h after micro-sized ZnO exposure than after nano-sized ZnO exposure. The difference in the effect size in levels of the neutrophils was statistically significant according to the univariate analysis. The adjusted OR of CRP and CC16 found in GEE analyses were comparable to those found with univariate evaluation.

### Sputum parameters

Nano- and micro-sized ZnO exposures led to a significant increase of MMP-9, whereas a significant increase was observed for TIMP-1 only after exposure to nano-sized ZnO and a not significant increase after micro-sized ZnO exposure (Table 4). In addition, increases in IL-8 values were observed, but these were not significant for either micro- or nano-sized ZnO particles. At the final examination, all three parameters (IL-8, MMP-9, TIMP-1) were decreased and were below the initial values. All other parameters (total cell number, total protein and neutrophils) were unaffected by ZnO exposures. The detailed results of the univariate analyses of all induced sputum parameters and further results of the GEE analyses are presented in the supplementary material (Table S1, S2).

**Table 4:** GEE analyses of selected sputum parameters considering ZnO particle size and time of sampling

Parameter	Time of sampling	ZnO [mg/m^3^]	OR^a^	OR 95% CI	*P* value
IL-8 [pg/mL]	22 after exposure	0	1.00		
	22 after exposure	2–micro	1.57	0.82–2.99	0.1715
	22 after exposure	2–nano	1.71	0.99–2.93	0.0528
	Final examination		0.51	0.35–0.76	**0.0007**
MMP-9 [ng/mL]	22 after exposure	0	1.00		
	22 after exposure	2–micro	2.90	1.59–5.29	**0.0005**
	22 after exposure	2–nano	2.84	1.59–5.07	**0.0004**
	Final examination		0.63	0.45–0.88	**0.0061**
TIMP-1 [ng/mL]	22 after exposure	0	1.00		
	22 after exposure	2–micro	1.66	0.94–2.92	0.0826
	22 after exposure	2–nano	1.97	1.25–3.10	**0.0033**
	Final examination		0.50	0.30–0.84	**0.0094**

^a^OR adjusted for ZnO exposure, time of sampling: 22 h after exposure, BMI, and total IgE. *P* values in bold are statistically significant

ZnO exposures had no effect on blood pressure, FeNO and all lung function parameters.

### Calculation of deposition rates of inhaled ZnO particles

**Fig. 6:**
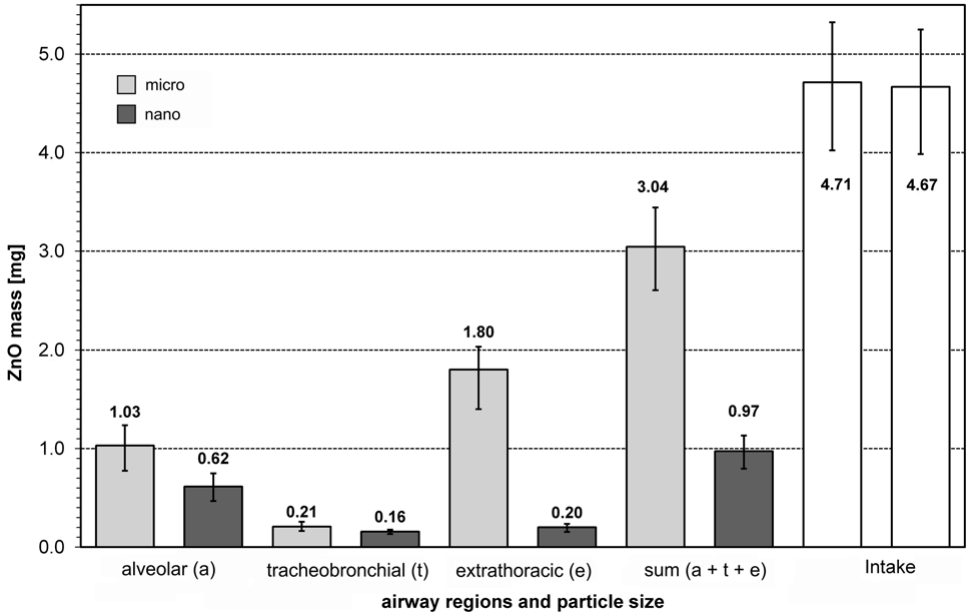
Calculated lung deposition efficiency of ZnO particles in dependence of airway regions (*a* alveolar region, *t* tracheobronchial region, *e* extrathoracic region) and particle size. Sum: total deposition (sum of *a*, *t* and *e*), Intake: Total inhaled mass of ZnO particles

Figure 6 shows the estimation of the masses of deposited ZnO particles of different airway regions according to particle sizes. Shown are the mean values of the deposited masses of all participants. The error bars represent the minimum and maximum values. The total inhaled mass of ZnO particles is also shown. The difference in deposition rates between micro- and nano-sized ZnO particles in the alveolar region was about 40%. The difference in the tracheobronchial region was only small, but in the extrathoracic region, the difference was large at 89%. The deposited masses from all airway compartments yielded about threefold higher deposition rates for micro-sized than for nano-sized particles while the difference between the inhaled total masses of micro- and nano-sized ZnO particles was negligible (< 1%).

## Discussion

Systemic effects after inhalation of micro-sized ZnO particles were more pronounced than effects of the nano-sized particles. This key result falsified our hypothesis. The most likely explanation might be the significant higher total and also specific deposition rates for micro-sized compared to nano-sized particles in all airway compartments. The most sensitive outcomes were the particle size-dependent changes in neutrophils and CC16 in blood. Especially the concentrations of serum CC16 showed a conspicuous time course (increase in CC16 concentrations after 22 h followed by significantly reduced concentrations 2 and 3 days after exposure). This course is observed for both particle sizes, but more pronounced for the micro-sized ZnO particles. No relevant effects were detectable after sham exposures, as shown in our previous study (Monsé et al. 2018, 2019).

The dose of ZnO administered (2.0 mg/m^3^ × 2 h) was derived from our previous study (Monsé et al. 2018) (1.0 mg/m^3^ × 4 h) to yield mild systemic inflammatory responses and to avoid adverse effects observed at the highest ZnO dose in that study (2.0 mg/m^3^ × 4 h). Blood and sputum parameters which showed effects in the earlier study (Monsé et al. 2018, 2019) were selected for this investigation. Parameters that were unaffected in the earlier and, therefore, excluded in the present study were Zn levels in blood and urine, IL-6, clotting factors prothrombin F 1.2, endothelial microparticles, fibrinogen, D-dimers (Monsé et al. 2018) as well as cardiovascular effects (Aweimer et al. 2020). The effect strength of the chosen ZnO dose was confirmed by mild body temperature increases in only two subjects after ZnO exposure. No relevant increases of questionnaire ratings and only mild responses were detected in blood and sputum parameters.

In our previous study (Monsé et al. 2018) with nano-sized ZnO particles and the only time point of examination 24 h after exposure, we could not show a significant influence on the CC16 concentration. In the present study with the two additional time points (blood collection two and three days after exposure), a significant decrease of the CC16 concentrations compared to sham exposure was observed at these time points, especially for micro-sized ZnO, whereas a slight but significant increase in CC16 concentrations was observed 22 h after exposure to micro-sized ZnO. Several studies have shown that the serum CC16 concentration, a well-validated marker of the lung epithelium barrier integrity, is decreased in subjects with chronic lung damage caused by tobacco smoke (Lam et al. 2018) and other air pollutants (Broeckaert et al. 2000a). Other studies (summarized e.g. by Provost et al. 2014) observed an increase after diverse environmental exposures, such as trichloroamine, among regular attendees of chlorinated indoor swimming pools, ambient ozone and particulated air pollution in elderly men. Especially in the study on ambient ozone exposure (Broeckaert et al. 2000b), blood samples were provided immediately before and after the exposure for the analysis of CC16. Therefore, it must be taken into account that the CC16 level in serum is not only influenced by a diurnal rhythm (Helleday et al. 2006), but also depends strongly on the time of blood collection after exposure. In the blood samples collected at the final time point of minimum of two weeks after the last exposure, the CC16 levels were not significantly different from the levels at baseline examination, indicating that there has been no permanent impairment of the lung epithelium barrier integrity.

No effects were reported in the only previous human inhalation study investigating different particle sizes at 0.5 mg/m^3^ ZnO (Beckett et al. 2005). However, in addition to the fourfold lower ZnO concentration, Beckett and co-workers performed the 2-h exposure without physical activity, which resulted in an approximately eightfold lower inhaled dose (using linear extrapolation) of ZnO compared to our previous study. Another difference was that these authors generated the larger ZnO particles by an agglomeration process of nano-sized ZnO particles and applied the particles via mask breathing. For technical reasons, a crystalline, micro-sized ZnO product was used in our study because a comparable agglomeration process was not feasible due to too large volume flows in our experimental whole body exposure setting. The two materials used in this study differ in a number of ways: (a) the way they are manufactured (which may impact on surface structure that could, for example, impact on dissolution behaviour); (b) the primary particle size (and potentially size distribution), which could again affect cellular uptake and dissolution in the lung; (c) the size of the aerosolised particles (which affects the degree and location of deposition); and (d) the morphology of the aerosol particles (i.e. commercial form agglomerates, whereas the pyrolysis-generated form aggregates that further agglomerate) which may also affect deposition and subsequent behaviour in the lung.

The hypothesis that nano-sized ZnO particles exert stronger effects than micro-sized ZnO particles was not confirmed. Stronger effects of nano-sized compared to micro-sized particles can be explained by the higher volume consumption of their agglomerates in the lysosomes of phagocytizing alveolar macrophages (Pauluhn 2011, 2014; Federal Institute for Occupational Safety and Health 2015). Different dissolution behaviour and particle surface area would as well explain stronger effects of nano-sized ZnO (Oberdörster et al. 2005). Nano-sized ZnO particles are expected to dissolve faster than the micro-sized particles and, thus, would lead to a faster influx of zinc ions and stronger initial effects. Larger surface area of nano-sized ZnO particles could as well result in a higher effect strength of the nano-sized ZnO. However, all these cannot explain the stronger health effects of micro-sized ZnO in human volunteers. In fact, the results of the particle-induced cell migration assay indicate that dissolution behaviour, specific surfaces area, morphology or possible chemical impurities do not contribute significantly to the cellular effects of the two ZnO particle species. Indeed there is experimental evidence that ZnO is not a GBP and its substance-specific mode of action is caused by the formation of zinc ions. Cho et al. (2011) suggested that the rapid pH-dependent dissolution of the ZnO particles in the phagosomes is the main cause of ZnO toxicity. This notion is supported by the relatively short half-life of the ZnO nanoparticles in the rat lung between 4.8 and 19.2 h (Pauluhn 2014; Hollinger et al. 1979). This corresponds to the time period from ZnO exposure to the observation of the systemic imflammatory effects in humans. With the help of the ICRP model (ICRP 1994) for a reference worker, with comparable physical load of our subjects, we are able to explain our results. The higher systemic inflammatory responses of the subjects of our study after inhalation of micro-sized ZnO particles were probably induced by the about threefold higher deposition efficiency of micro-sized ZnO particles and, therefore, the higher zinc ion release in the respiratory tract in comparison of nano-sized ZnO particles. Obviously, the effects of different dissolution behavior, particle surface area or differences in the morphology between ZnO nano- and microparticles are too small to have an visible effect in our controlled human study compared to the different deposition efficiency.

One additional weakness of the present study is that the recording of systemic and local effect parameters is limited to certain time points due to constraints of the study design. Missing of effects might be caused by an earlier or later increase compared to the sampling times. The blood parameters, however, consistently showed that the effects were more pronounced after inhalation of micro-sized ZnO particles. In addition, inflammatory effects in induced sputum showed no clear differences between nano- and micro-sized ZnO. Similarly, in our previous study with three ZnO concentrations, dose-dependency of the effects was found for inflammatory markers in blood, but not in induced sputum (Monsé et al. 2019). According to our data, sputum is less suitable to assess ZnO-induced inflammatory effects quantitatively. As discussed earlier, the reasons for this discrepancy may be manifold, but remain largely unknown (Monsé et al. 2019). Another weakness of this study is the lack of SEM images of the airborne ZnO particles. The imaging of the micro-sized ZnO (Fig. 2) also showed particles in the nanometer range, but according to the APS and SMPS measurements, they were not present in the atmosphere of the ExpoLab. Presumably, agglomerates were formed during the aerosolisation process, to which the nano-sized fraction stuck by adhesion. Further experiments are currently being conducted in this regard. A SEM image of airborne ZnO nanoparticles generated by the flame generator used in this study was shown earlier (Monsé et al. 2014).

The strength of this study is the lack of effects after sham exposures (0 mg/m^3^ ZnO). Many control conditions were performed without ZnO exposure (blood parameter: baseline examination, before exposures with examinations 22 h after, as well as two and three days after sham exposure and final examination). For the sputum parameters, three control scenarios (baseline examination, 22 h after sham exposure and final examination) were available. Thus, accidental variabilities were minimized. Furthermore, our data clearly demonstrate that all observed effects were reversible as shown at the final examination about two weeks after the last ZnO exposure.

Reference values for most of the effect parameters in this study do not exist or show large inter-individual variability. Thus, descriptive analyses with respect to reference values and interpretations of the magnitudes of the effects were not considered. Instead, univariate group comparisons (before/22 h after exposure) and additional multivariate GEE analyses were used. The number of 16 subjects was sufficient, as they were exposed twice to ZnO and sham serving as their own control. Since the results presented here show statistically significant changes, we have refrained from presenting the power calculation we carried out in advance.

In our previous study, a No Effect Exposure Level (NOEL) was suggested between 0.5 and 1 mg/m^3^ (Monsé et al. 2018). This derivation was based on data from nano-sized ZnO particle exposure experiments. Zinc-containing fumes or dust at workplaces are not only nano-sized, but also, e.g. due to agglomeration processes, micro-sized particles can be found. However, the hazard for workers exposed to nano-sized ZnO is not stronger than for subjects exposed to micro-sized ZnO particles according to the data presented here. Conversely, the investigation of whether the NOEL of micro-sized ZnO particles is even lower than that of ZnO nanoparticles can only be addressed with a further study with different ZnO concentrations.

## Conclusion

In summary, this study was able to demonstrate stronger effects after inhalation of micro-sized than nano-sized ZnO particles in human volunteers. This can be explained by the higher deposition efficiency of micro-sized ZnO particles in the respiratory tract and a substance-specific mode of action, most likely caused by the formation of zinc ions. These results should be taken into account when establishing an occupational exposure limit value for zinc-containing fumes.

## Electronic supplementary material

Below is the link to the electronic supplementary material.

Supplementary file1 (PDF 236 kb)
