# Supplementary material for: Health effects after inhalation of micro- and nano-sized zinc oxide particles in human volunteers
Source: Arch Toxicol. 2020 Oct 1;95(1):53–65. doi: 10.1007/s00204-020-02923-y (PMC7811523; doi:10.1007/s00204-020-02923-y)
Supplement: Supplementary file 1 — Supplementary file1 (PDF 236 kb) [file 204_2020_2923_MOESM1_ESM.pdf]

Comparing health effects after inhalation of micro- and nano-sized zinc oxide particles in human volunteers

Archive of Toxicology

Christian Monsé, Monika Raulf, Birger Jettkant, Vera van Kampen, Benjamin Kendzia, Leonie Schürmeyer, Christoph Edzard Seifert, Eike-Maximilian Marek, Götz Westphal, Nina Rosenkranz, Rolf Merget, Thomas Brüning, Jürgen Bünger

Institute for Prevention and Occupational Medicine of the German Social Accident Insurance, Institute of the Ruhr-Universität Bochum (IPA), Bürkle-de-la-Camp-Platz 1, 44789 Bochum, Germany, E-mail: monse@ipa-dguv.de, fon: +49 (0)30 130014129, fax: +49 (0)30 13001864129.

Table S1: Acute effects of ZnO exposure on blood and induced sputum parameters at different time points [median (minimum-maximum)]

**Parameters**	**Basis examination**	**ZnO [mg/m^3]**	**Directly before exposure**	**22h after exposure**	**2 days after exposure**	**3 days after exposure**	**Final examination**
**Blood**							
Leukocytes [1/nL]		0	5.95 (4.40-8.10)	5.55 (3.60-8.30)	5.55 (4.10-8.60)	6.50 (3.80-10.40)	
	5.65 (4.00-9.30)	2 - nano	5.45 (4.40-8.70)	7.10 (4.60-9.50)	5.30 (3.90-7.10)	5.35 (4.10-7.70)	5.40 (3.50-8.00)
		2 - micro	5.10 (4.30-10.50)	8.90 (5.40-12.80)	5.35 (3.90-13.00)	5.60 (3.80-10.20)	
Neutrophils [%]		0	55 (42-70)	54 (43-66)	53 (42-74)	56 (34-74)	
	56 (43-77)	2 - nano	55 (42-67)	64 (53-72)	52 (42-64)	52 (45-67)	53 (40-64)
		2 - micro	53 (43-73)	71 (61-76)	55 (41-83)	58 (42-79)	

Neutrophils [1/nL]		0	3.23 (2.21-5.70)	3.07 (1.63-5.50)	2.82 (1.92-6.40)	3.25 (1.86-7.68)	
	3.18 (1.95-7.18)	2 - nano	2.90 (2.19-5.78)	4.25 (2.73-6.64)	2.75 (1.85-4.48)	2.72 (2.04-5.13)	2.94 (1.39-5.08)
		2 - micro	2.63 (2.10-7.60)	5.94 (3.64-9.71)	2.69 (1.86-10.74)	3.00 (1.95-7.65)	
Lymphocytes [%]		0	30 (18-42)	35 (23-43)	36 (18-45)	32 (16-59)	
	33 (15-41)	2 - nano	33 (22-42)	26 (19-38)	36 (25-43)	35 (23-45)	36 (24-50)
		2 - micro	34 (17-46)	21 (15-30)	32 (11-47)	31 (13-43)	
Lymphocytes [1/nL]		0	1.84 (0.92-2.68)	1.84 (1.34-2.54)	1.71 (1.24-2.72)	1.81 (1.17-3.78)	
	1.61 (1.21-2.98)	2 - nano	1.89 (1.21-2.76)	1.82 (1.24-2.64)	1.69 (1.33-2.50)	1.78 (1.32-2.97)	1.90 (1.32-2.18)
		2 - micro	1.79 (1.22-2.55)	1.70 (1.10-2.42)	1.72 (1.29-2.52)	1.73 (1.27-2.95)	
Monocytes [%]		0	8 (6-11)	9 (6-11)	8 (5-11)	9 (5-11)	
	9 (6-14)	2 - nano	9 (6-12)	7 (4-10)	9 (5-12)	8 (5-12)	8 (5-12)
		2 - micro	9 (5-12)	7 (5-12)	8 (4-12)	9 (5-18)	
Monocytes [1/nL]		0	0.52 (0.34-0.88)	0.47 (0.27-0.82)	0.45 (0.33-0.74)	0.49 (0.34-0.85)	
	0.52 (0.28-1.06)	2 - nano	0.47 (0.31-0.73)	0.53 (0.31-0.74)	0.45 (0.28-0.82)	0.46 (0.30-0.81)	0.45 (0.26-0.86)
		2 - micro	0.47 (0.37-0.80)	0.57 (0.38-1.12)	0.45 (0.27-0.78)	0.50 (0.31-0.87)	

Thrombocytes [1/nL]		0	215 (172-316)	221 (163-326)	225 (168-338)	237 (182-332)	
	237 (165-311)	2 - nano	229 (168-345)	234 (170-327)	226 (169-340)	233 (167-348)	231 (178-321)
		2 - micro	234 (178-359)	218 (170-334)	230 (176-367)	232 (180-359)	
Erythrocytes [1/nL]		0	4.55 (4.00-5.50)	4.60 (4.00-5.20)	4.60 (3.90-5.40)	4.70 (3.90-5.70)	
	4.60 (4.10-5.60)	2 - nano	4.60 (4.20-5.40)	4.55 (4.10-5.30)	4.60 (4.20-5.20)	4.65 (4.20-5.40)	4.65 (4.10-5.40)
		2 - micro	4.65 (4.10-5.40)	4.50 (4.00-5.40)	4.50 (4.30-5.40)	4.60 (4.20-5.50)	
CRP [µg/mL]		0	1.13 (0.07-9.28)	0.77 (0.08-9.34)	0.81 (0-11.19)	0.61 (0-25.54)	
	0.53 (0.04-4.06)	2 - nano	0.37 (0-5.65)	1.87 (0.02-11.03)	1.69 (0.05-11.67)	1.03 (0-7.37)	0.43 (0-3.84)
		2 - micro	0.84 (0-5.46)	2.23 (0.06-8.43)	3.10 (0.03-13.84)	1.74 (0-13.41)	
CC16 [ng/mL]		0	9.37 (5.87-13.86)	10.04 (5.61-15.68)	9.75 (5.26-12.86)	10.14 (5.95-12.45)	
	9.34 (5.71-14.12)	2 - nano	10.00 (6.48-12.70)	11.11 (7.50-17.32)	7.88 (4.86-10.06)	8.97 (5.69-12.27)	9.25 (5.62-14.42)
		2 - micro	10.05 (5.85-16.55)	12.21 (7.80-17.95)	7.76 (4.35-11.30)	8.10 (6.07-10.52)	
SAA [ng/mL]		0	14134 (<1880-125109)	9949 (2341-152914)	11296 (2695-224552)	8317 (2314-253235)	
	11353 (<1880-74913)	2 - nano	11653 (<1880-72687)	42471 (<1880-210008)	29550 (<1880-99339)	11653 (<1880-72687)	10947 (<1880-41150)
		2 - micro	11572 (<1880-86546)	31595 (2357-63553)	49813 (2532-137718)	18159 (3437-87063)	

**Induced sputum**							
IL-8<br>[pg/mL]		0		1400<br>(252-5195)			
	1021<br>(206-4327)	2 - nano		1745<br>(322-21645)			1110<br>(150-17119)
		2 - micro		1532<br>(233-17590)			
MMP-9<br>[ng/mL]		0		87<br>(13-307)			
	106<br>(18-344)	2 - nano		98<br>(32-3288)			61<br>(15-480)
		2 - micro		128<br>(19-1382)			
TIMP-1<br>[ng/mL]		0		6.84<br>(1.15-67.70)			
	7.87<br>(0.13-57.27)	2 - nano		8.21<br>(1.69-124.68)			5.62<br>(0.52-120.84)
		2 - micro		7.57<br>(1.20-162.64)			

Table S2: Influence of ZnO exposure and time of sampling on blood parameters

Parameter	**Time of sampling**	**ZnO [mg/m³]**	**OR[1]**	**OR 95% CI**	**P value**
**Blood**					
Leukocytes [1/nL]					
	Before exposure	0	1.00		
	22 after exposure	2 - micro	1.60	1.38 – 1.85	<0.0001
	22 after exposure	2 - nano	1.31	1.18 – 1.46	<0.0001
	2 days after exposure	2 - micro	1.10	0.94 – 1.29	0.2502
	2 days after exposure	2 - nano	0.96	0.84 – 1.10	0.5438
	3 days after exposure	2 - micro	0.98	0.85 – 1.14	0.7998
	3 days after exposure	2 - nano	0.90	0.79 – 1.03	0.1255
	Final examination	0	1.03	0.89 – 1.20	0.6617
Neutrophils [%]	Before exposure	0	1.00		
	22 after exposure	2 - micro	1.32	1.23 – 1.42	<0.0001
	22 after exposure	2 - nano	1.20	1.12 – 1.28	<0.0001
	2 days after exposure	2 - micro	1.06	0.96 – 1.16	0.2485
	2 days after exposure	2 - nano	1.03	0.94 – 1.11	0.5533
	3 days after exposure	2 - micro	1.07	0.96 – 1.18	0.2232
	3 days after exposure	2 - nano	1.02	0.91 – 1.14	0.7576
	Final examination	0	0.92	0.82 – 1.03	0.0778
Lymphocytes [%]	Before exposure	0	1.00		
	22 after exposure	2 - micro	0.59	0.52 – 0.68	<0.0001
	22 after exposure	2 - nano	0.74	0.65 – 0.83	<0.0001
	2 days after exposure	2 - micro	0.88	0.74 – 1.05	0.1635
	2 days after exposure	2 - nano	0.94	0.81 – 1.10	0.4357
	3 days after exposure	2 - micro	0.91	0.81 – 1.02	0.0927
	3 days after exposure	2 - nano	0.98	0.82 – 1.16	0.7955

	Final examination	0	0.97	0.80 – 1.13	0.6333
Lymphocytes [1/nL]	Before exposure	0	1.00		
	22 after exposure	2 - micro	0.95	0.86 – 1.04	0.2626
	22 after exposure	2 - nano	0.96	0.82 – 1.12	0.6217
	2 days after exposure	2 - micro	0.97	0.88 – 1.08	0.6249
	2 days after exposure	2 - nano	0.90	0.77 – 1.06	0.2012
	3 days after exposure	2 - micro	0.89	0.77 – 1.04	0.1410
	3 days after exposure	2 - nano	0.89	0.75 – 1.05	0.1591
	Final examination	0	1.04	0.91 – 1.18	0.7235
Monocytes [%]	Before exposure	0			
	22 after exposure	2 - micro	0.82	0.72 – 0.93	0.0017
	22 after exposure	2 - nano	0.86	0.77 – 0.95	0.0042
	2 days after exposure	2 - micro	0.92	0.81 – 1.03	0.1575
	2 days after exposure	2 - nano	1.03	0.93 – 1.14	0.5471
	3 days after exposure	2 - micro	0.99	0.82 – 1.20	0.9360
	3 days after exposure	2 - nano	1.03	0.90 – 1.18	0.6668
	Final examination	0	0.97	0.79 – 1.17	0.7147
Thrombocytes [1/nL]	Before exposure	0	1.00		
	22 after exposure	2 - micro	0.97	0.93 – 1.01	0.0944
	22 after exposure	2 - nano	0.99	0.96 – 1.02	0.4895
	2 days after exposure	2 - micro	0.99	0.94 – 1.04	0.6376
	2 days after exposure	2 - nano	0.97	0.93 – 1.00	0.0691
	3 days after exposure	2 - micro	0.98	0.93 – 1.03	0.4685
	3 days after exposure	2 - nano	0.94	0.90 – 0.99	0.0124
	Final examination	0	1.03	0.99 – 1.08	0.3356
Erythrocytes [1/nL]	Before exposure	0	1.00		
	22 after exposure	2 - micro	0.99	0.97 – 1.02	0.5280

	22 after exposure	2 - nano	0.99	0.97 – 1.01	0.1909
	2 days after exposure	2 - micro	1.00	0.97 – 1.03	0.9327
	2 days after exposure	2 - nano	1.00	0.98 – 1.02	0.8450
	3 days after exposure	2 - micro	1.00	0.96 – 1.04	0.9692
	3 days after exposure	2 - nano	1.00	0.97 – 1.04	0.9172
	Final examination	0	1.01	0.98 – 1.05	0.8878

[1]: Odds ratios adjusted for ZnO exposure, Time of sampling, BMI, and total IgE.

PICMA:

The particle induced migration assay (PICMA) models inflammatory reactions in the lung. Lung inflammation is characterized by the accumulation of inflammatory cells. In the first line of defense macrophages are recruited. If the macrophages are not able to cope with the pathogens, neutrophils are additionally recruited and activated. This is also true for particle exposure.

Both reactions can be modelled in vitro: Supernatants of particles challenged NR8383 macrophages attract differentiated HL-60 cells as a model for neutrophils (Westphal et al. 2015) as well as unchallenged macrophages (Schremmer et al. 2016). Kinetic investigations show that 16 hours incubation yielded the strongest effects in terms of cell migration and the parallel formation of inflammatory markers (Schremmer et al. 2016).

The primarily intended use of the human tumor cell line THP-1 was not successful, since these cells did not lead to a particle induced cell migration (Westphal et al. 2015). NR8383 cells are derived from healthy rat lung alveolar macrophages and share lots of properties with physiological macrophages (Helmke et al. 1987). The particle induced cell migration is a good reflection of the strength of the particles including fibers in vivo (Westphal et al. 2015, 2019).

Helmke RJ, Boyd RL, German VF, Mangos JA (1987) From growth factor dependence to growth factor responsiveness: the genesis of an alveolar macrophage cell line. In Vitro Cell Dev Biol 23:567-574

Westphal GA, Schremmer I, Rostek A, Loza K, Rosenkranz N, Brüning T, Epple M, Bünger J (2015) Particle-induced cell migration assay (PICMA): A new in vitro assay for inflammatory particle effects based on permanent cell lines. Toxicol In Vitro 29:997-1005

Westphal GA, Rosenkranz N, Brik A, Weber D, Föhring I, Monsé C, Kaiser N, Hellack B, Mattenklott M, Brüning T, Johnen G, Bünger J (2019) Multi-walled carbon nanotubes induce stronger migration of inflammatory cells in vitro than asbestos or granular particles but a similar pattern of inflammatory mediators. Toxicol In Vitro 58:215-223

Schremmer I, Brik A, Weber DG, Rosenkranz N, Rostek A, Loza K, Brüning T, Johnen G, Epple M, Bünger J, Westphal GA (2016) Kinetics of chemotaxis, cytokine, and chemokine release of NR8383 macrophages after exposure to inflammatory and inert granular insoluble particles. Toxicol Lett 263:68-75
